# Strategies to overcome vaccine hesitancy: a systematic review

**DOI:** 10.1186/s13643-022-01941-4

**Published:** 2022-04-26

**Authors:** Prem Singh, Pritu Dhalaria, Satabdi Kashyap, Gopal Krishna Soni, Partha Nandi, Shreeparna Ghosh, Mrinal Kar Mohapatra, Apurva Rastogi, Divya Prakash

**Affiliations:** 1 Immunization Technical Support Unit, Ministry of Health & Family Welfare, Government of India, New Delhi, India; 2 New Delhi, India; 3 Department of Community Medicine, College of Medicine, University of Bisha - Ministry of Higher Education, Bisha, Kingdom of Saudi Arabia; 4 Institute of Medical Sciences and SUMS Hospital, Bhubaneswar, Odisha India

**Keywords:** Global health, Vaccine hesitancy, Immunization, Vaccines, Vaccination, Vaccine refusal

## Abstract

**Background:**

Vaccination, albeit a necessity in the prevention of infectious diseases, requires appropriate strategies for addressing vaccine hesitancy at an individual and community level. However, there remains a glaring scarcity of available literature in that regard. Therefore, this review aims to scrutinize globally tested interventions to increase the vaccination uptake by addressing vaccine hesitancy at various stages of these interventions across the globe and help policy makers in implementing appropriate strategies to address the issue.

**Methods:**

A systematic review of descriptive and analytic studies was conducted using specific key word searches to identify literature containing information about interventions directed at vaccine hesitancy. The search was done using PubMed, Global Health, and Science Direct databases. Data extraction was based on study characteristics such as author details; study design; and type, duration, and outcome of an intervention.

**Results:**

A total of 105 studies were identified of which 33 studies were included in the final review. Community-based interventions, monetary incentives, and technology-based health literacy demonstrated significant improvement in the utilization of immunization services. On the other hand, media-based intervention studies did not bring about a desired change in overcoming vaccine hesitancy.

**Conclusion:**

This study indicates that the strategies should be based on the need and reasons for vaccine hesitancy for the targeted population. A multidimensional approach involving community members, families, and individuals is required to address this challenging issue.

**Supplementary Information:**

The online version contains supplementary material available at 10.1186/s13643-022-01941-4.

## Background

Vaccines have always been one of the most innocuous and effective approaches for the prevention of many infectious diseases [1]. In spite of this, vaccine-preventable diseases are still widespread. In the preceding years, there have been outbreaks of infectious diseases in many parts of the world regardless of having effective vaccines against such diseases. The plausible reason for it could be “vaccine hesitancy” [2].

Vaccine hesitancy refers to a delay in acceptance or refusal of vaccination despite availability of vaccination services [3]. Against the backdrop of a large number of unimmunized children globally and frequent outbreaks of vaccine-preventable diseases [4], WHO has listed vaccine hesitancy as one of the top ten global health threats in 2019 [5] and has drawn major concerns across the world due to increase and resurgence of vaccine-preventable diseases. The reasons of reluctance or refusal are complex varying across time, place, specific type of vaccines [6, 7], and context-specific such as related to confidence, convenience, and complacency. Similarly, multiple factors such as religious beliefs, geographic barriers, parent-provider relationship, perceived risk of adverse events following immunization (AEFI), lack of knowledge about vaccination, and disease risk perception give rise to vaccine hesitancy [8]. A survey conducted by WHO and UNICEF showed that vaccine hesitancy emerged a decade ago [9]. However, it has gained attention due to the current changing scientific, cultural, medico-legal, and media environments, despite all the efforts made to increase the awareness and increase the vaccines uptake. The trend has been realized in several countries across the world including the UK, USA, and India [10]. This has triggered global researchers to understand the determinants of this emerging issue throughout the world. One of the reviews conducted by Jarrett et al. (2015) on similar background and methodology have conducted their review on the basis of three broad theme {dialogue-based, incentive-based (non-financial), reminder/recall-based} have some of the shortcomings. The study did not mention technology-based health literacy as well as incentive based on financial aspect in their review. The study also includes grey literature in their review which arises the potential literature bias in the review [11].

Various strategies such as community activity by community health workers and medical interns, monetary incentives, and educational videos as well as media-based approach have been piloted and evaluated in diverse settings to understand their impact on reducing the vaccine hesitancy. However, there is a paucity of critical synthesis of all these interventions across the globe and contextual summarization to guide program managers and policy makers in implementing appropriate strategies to address vaccine hesitancy. Therefore, this review aims to analyze globally tested interventions to increase the vaccination uptake by addressing the issues through globally tested interventions for people with different degrees of vaccine hesitancy.

## Methods

**Fig. 1:**
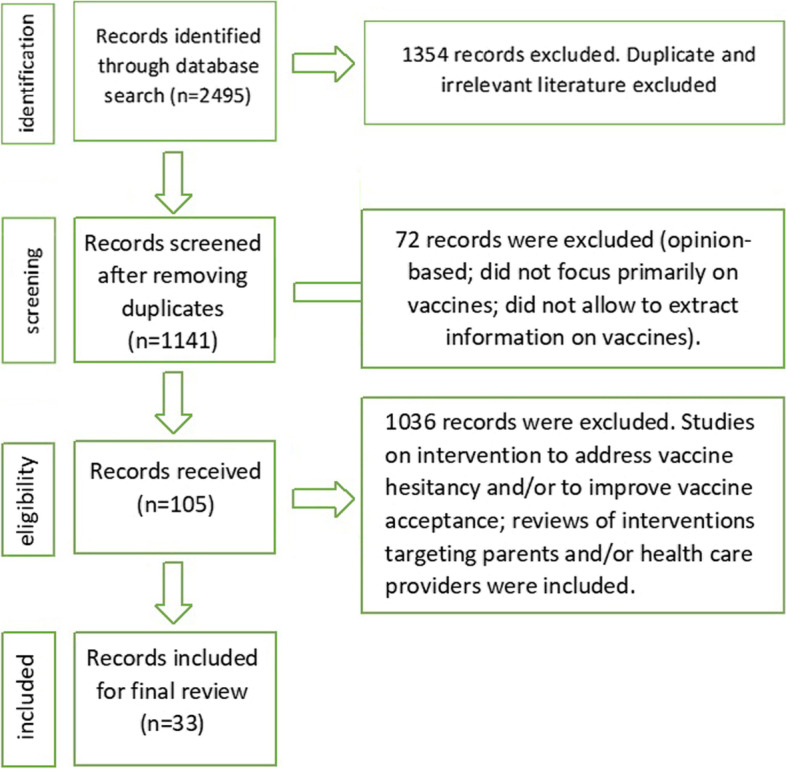
Literature review data synthesis flowchart

This systematic review was reported in line with the quality requirements of the PRISMA reporting guideline, from June to September 2019 and the flow chart has been mentioned as Fig. 1 for understanding the method followed [12]. The checklist of PRISMA reporting guideline has also been added as Additional Document.

A search was conducted in the PubMed, Global Health, and Science Direct electronic databases to identify peer-reviewed literature. Search was not restricted to any time period and included literature search for title, abstract, and full-text in English language only.

### Search strategy

The search strategy was set up using database-specific vocabularies. The literature search was conducted using the keywords “immunization,” “vaccine,” “vaccination,” “vaccine strategy,” “vaccine intervention,” “vaccine hesitant,” “vaccine hesitancy,” “vaccine refusal,,” “trust in vaccination,” “vaccine confidence,” “vaccine resistance,” “vaccine impact,” “vaccine concern,” “vaccine rejection,” and “vaccine side effects” using “AND” and “OR” operators.

### Inclusion and exclusion criteria

While searching for vaccination strategies, we considered universally recommended vaccines for children, adolescents, and adults such as diphtheria, tetanus, pertussis, poliomyelitis, hepatitis B, tuberculosis (BCG vaccine) measles, mumps, rubella, hemophilus influenza B (Hib), varicella, pneumococcal vaccine, meningococcal vaccine, human papillomavirus (HPV), oral polio vaccine, and seasonal influenza vaccine. Based on the objective, we included interventions that were targeted towards addressing vaccine hesitancy among parents and caregivers. For review, descriptive and analytical studies that described the effect of strategies on addressing vaccine hesitancy were included.

Studies that were opinion-based or did not focus primarily on populations eligible to receive vaccine or their parents or that did not allow the authors to extract information on vaccination were excluded from our analysis.

### Study selection process

Two researchers independently reviewed the identified studies for eligibility using a two-step process. In the first step, title, abstract, and keywords were screened to segregate the eligible studies followed by a full-text retrieval and screening. Similarly, data extraction was performed independently by two researchers and unmatched studies were included or excluded in consensus with a third researcher.

### Data extraction and synthesis

Data extraction included study characteristics such as (1) author, year, journal, study design, study setting, study period, and study population; (2) the vaccines considered; (3) information about the intervention being studied such as type of intervention and duration of the intervention; and (4) information on follow-up time, analysis performed, and outcomes of interest.

**Fig. 2:**
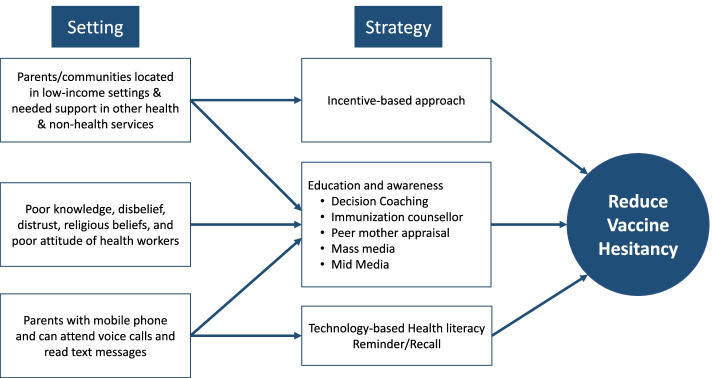
Strategies to remove a vaccine hesitancy

We categorized the review under four broad themes, i.e., community health training, incentive-based approach; technology-based health literacy; and media engagement using participants, interventions, comparisons, outcomes, and study design (PICO) strategy (Fig. 2) [13].

1. *Community health trainings:* It included community health information dissemination through health workers, mobilizers, medical officers; social mobilization through medical interns, prominent religious leaders; and knowledge- and experience-sharing by influential women from the community to accelerate vaccine uptake [13].
2. *Incentive-based approach:* It involved incentives to encourage parents to immunize their children, including provision of food, other goods, and certificates of recognition or monetary support to encourage vaccination [13].
3. *Technology-based health literacy:* It involved use of technology in informing beneficiaries through various modern age-technologies such as mobile phone. Activities in this category included mobile phone recall text messages in local languages, pictorial messages, and automated phone calls or interactive voice recording for spreading awareness [13].
4. *Media engagement:* Mobilization through various campaigns and platforms such as radio, TV, and print media should feature concise, easily understood public service announcements by national public figures, well-known and authoritative local representatives, and representative members of the target population [13].

### Critical appraisal

The Effective Public Health Practice Project (EPHPP) quality assessment tool for quantitative studies was applied to determine the risk of bias in all eligible studies [14]. Literature screening and data extraction piloting was done on five documents by all three reviewers to standardize the review and data extraction process. Furthermore, disagreements during review were resolved by consensus.

## Results

The search identified 2495 peer-reviewed articles. After removing duplicates, 1141 articles were screened using title, abstract, and keywords, which excluded 1036 papers leaving 105 full-text papers for review. Of these, 33 were evaluated and described. Among the evaluated peer-reviewed literature, nine were related to community health training’s theme [15–23], five were related to incentive-based approach [24–28], eight were related to technology-based health literacy [18, 29–35], and eleven were related to media engagement [36–46] (Tables 1 and 2).

**Table 1:** A descriptive summary of the characteristics of the included studies

Author	Study type	Name of country	Study setting	Participants	Interventions	Risk of bias score
Oche et al., 2011 [15]	Controlled community trial	Nigeria	Town with the vast majority of the population largely farmers and illiterates	Mothers of children aged 0 to 23 months	Community health training	6/10
Brugha et al., 1996 [16]	Controlled trial	Ghana	Town where regular immunization services were available	Mothers of 12–18-month-old children	Community health training	7/10
Zhang et al., 201 9[36]	Cross-sectional	Australia	Nationally representative sample	Parents with at least one child under 5 years	Media engagement	4/10
Rahman et al., 2013 [17]	Pre-post interventions without control	Iraq	District with both rural and urban population	Villages with a DPT 3 coverage rate < 20% and 15–24 infants below 1 year	Community health training	5/10
Williams et al., 2019 [47]	Cross-sectional	USA	Urban geographic area	Religious organizations with at least one religious leader or equivalent located in Denver county	Community health training	NA
Nasiru et al., 2012 [21]	Pre-post interventions without control	Nigeria	Local council with high reported cases of polio disease and very low vaccination uptake	Children under the age of 5	Community health training	7/10
Ofstead et al., 2013 [22]	Pre-post interventions with control	USA	Manufacturing corporation	Full-time employees and their dependents	Community health training	6/10
Ansari et al., 2007 [29]	Pre-post	India	High risk urban areas	High-risk urban areas	Technology-based health literacy	7/10
Usman et al., 2011 [23]	Randomized controlled trial	Pakistan	Rural EPI centers	All children visiting the selected EPI centers for DTP1	Community health training	9/10
Williams et al., 2013 [18]	Cluster-randomized controlled trial	USA	Private pediatric practices in urban area	Parent with a full-term infant less than 30 days old	Technology-based health literacy	9/10
Maltezou et al., 2009 [30]	Cross-sectional	Greece	Public hospitals	Greek public hospitals	Technology-based health literacy	6/10
Mouzoon, M. et al., 2010 [24]	Retrospective study	USA	A large multispecialty medical organization	Pregnant women and healthcare workers	Incentive based approach	8/10
Fiks, A.G et al., 2013 [31]	Cluster-randomized controlled trial	USA	Urban primary care practices	Girls 11 through 17 years of age due for at least 1 dose of the HPV vaccine	Technology-based health literacy	5/10
Spleen, A.M, et al., 2011 [19]	Pre-post	USA	Rural population with high poverty rates, high unemployment rates, low access to healthcare, and excess cancer burden, including cervical cancer	Parents of daughters age 9–17 years	Community health training	7/10
Muehleisen et al., 2007 [32]	Pre-post with control	Switzerland	Hospital in urban setting	Children aged 61 days to 17 years	Technology-based health literacy	7/10
Banerjee et al., 2010 [25]	Cluster-randomized controlled trial	India	Rural Rajasthan	Children aged 1–3 years	Incentive-based approach	9/10
Barham et al., 2008 [26]	Cluster-randomized controlled trial	The Republic of Nicaragua	Rural	Children 12–23-month-old and above	Incentive-based approach	7/10
Stitzer,M.L, et al. 2009 [27]	Randomized controlled trial	USA	General Hospital	Individual aged 18–64 years	Incentive-based approach	7/10
Robertson et al., 2013 [28]	Cluster-randomized trial	Zimbabwe	Four socioeconomic strata were selected: subsistence farming areas, roadside trading settlements, agricultural estates, and small towns	households with children younger than 18 years	Incentive-based approach	8/10
Stockwell et al., 2012 [37]	Two randomized controlled trials	USA	Urban, low-income population	Parents with children aged 11 to 18 years and families with a child aged 7 to 22 months lacking 1Hib dose	Media engagement	8/10
Milkman et al., 2011 [38]	Randomized controlled trial	USA	A large firm	Employees	Media engagement	8/10
Lemstra,M. et al. 2011 [40]	Cluster-randomized trial	Canada	Low-income setting	Parents of children who were behind in MMR immunizations	Media engagement	8/10
Clark et al., 2015 [41]	Internet-based cross-sectional survey	USA	Nationally representative sample	Parents of children 0 to 17 years of age	Media engagement (preferred mode of communication)	8/10
Kharbanda et al., 2009 [42]	Qualitative evaluation	USA	Three urban community health centers and two private practices in New York City	Parents with at least 1 child aged 10 to 19 years	Media engagement	8/10
Ahlers-Schmidt et al., 2010 [33]	Formative survey	USA	Low-income setting	Parents with children under 6 years of age at a Midwestern Pediatric Residency clinic	Technology-based health literacy	6/10
Hofstetter et al., 2013 [43]	Cross-sectional study	USA	Urban setting	Parents of 6–59-month-old children and providers	Media engagement (preferred recalled reminder mode)	7/10
Lau et al., 2012 [20]	Randomized controlled trial, cross-sectional study	Australia, Nigeria	University urban setting	University students and staff. Mothers and their infants aged 0–3 months	Community health training	9/10
Brown et al., 2017 [44]	Cross-sectional study	Nigeria	Urban and sub-urban community health facility	Mothers of infants	Media engagement (preferred recalled reminder mode)	6/10
Saville et.al, 2014 [45]	Cross-sectional, randomized, controlled trial	USA, Australia	Both urban and rural university	Parents of children 19–35-month-old University students and staff	Media engagement (preferred recalled reminder mode)	6/10
Cates et al., 2011 [34]	Assessment	4 North Carolina counties	Rural area	Mothers of girls aged 11–12	Media engagement (preferred recalled reminder mode)	6/10
Pandey et al., 2011 [35]	Cross-sectional	India	Medical school	Students of medical school	Technology-based health literacy	6/10
Garcia-Dia, 2017 [46]	Case-control study	Philippines	Rural setting	Parents of the 12–24 months children	Media-based approach	6/10
Moniz et al., 2013 [39]	Randomized controlled trial	USA	Outpatient clinic	Obstetric patients at less than 28 weeks of gestation pending the flu shot	Media-based approach	8/10

**Table 2:** A descriptive summary of the target vaccine, reason for hesitancy, outcomes, and limitations for each strategy

Author	Duration of study	Target vaccine	Reason for vaccine hesitancy	Outcome of interventions	Limitations of the study
**Community health training**					
Oche et al., 2011 [15]	9 months	DPT3	Low level of knowledge among mothers and poor attitude of health workers	Improved program acceptance and immunization services	Cost of services, availability of vaccines not considered
Brugha et al., 1996 [16]	8 months	BCG; poliovirus, DPT3, measles	Lack of awareness	Improvement of immunization coverage through community health training.	Contamination of control group
Rahman et al., 2013 [17]	6 months	DPT1, DPT2, DPT3, Measles	Lack of information/motivation	Vaccination coverage rates improved in intervention villages	Study restricted to a tribe influenced by peer-leader
Williams et al., 2019 [47]	5 months	Influenza	Religious beliefs/attitude	No significant outcome	Small study size
Nasiru et al., 2012 [21]	6 months	Polio vaccine	Attitude/misinformation	Effective communication and polio outreach campaigns-increased vaccine uptake	Population dynamics not considered
Ofstead et al., 2013 [22]	3 months	Influenza	Misconceptions	Substantial increase in vaccination rate	No psychometric evaluation
Usman et al., 2011 [23]	90 days	DTP	Lack of knowledge	Infant vaccination increased	Lack of complete follow-up
Spleen et al., 2011 [19]	1 year	HPV vaccine	Lack of parental attitude/knowledge	Increased vaccine acceptability	Study limited to small parent sub-group
Lau et al., 2012 [20]	6 months	Influenza	Lack of knowledge	Improved uptake of influenza vaccination and utilization of health services	Seasonal variations of influenza not considered.
**Incentive-based approach**					
Mouzoon et al., 2010 [24]	6 years	Influenza	Lack of familiarity or comfort with vaccination in pregnancy	Vaccination acceptability increased in pregnant females	Lack of baseline data
Banerjee et al., 2010 [25]	18 months	BCG, DPT, oral polio vaccines, measles	Lack of awareness	Increased uptake of immunization services.	Not a blinded study
Stitzer et al., 2009 [27]	6 months	HBV	Negligence	Motivation leading to attending vaccination sessions	Small sample size, homogeneity of sample
Barham et al., 2008 [26]	2 years	BCG, MCV, OPV3, DPT3	Lack of finance and motivation	Vaccination coverage increased dramatically	Proximity to availability of vaccine to study group not considered
Robertson et al., 2013 [28]	1 year	Childhood vaccination	Lack of motivation	No increase in vaccination uptake	Short intervention period
**Technology-based health literacy**					
Ansari et al., 2007 [29]	1-day study	Polio vaccine	Misguided information/rumors	Correct health education leading to vaccine acceptance	Other parameters and lack of existing immunization not considered
Williams et al., 2013 [18]	2 months	Pertussis, varicella, pneumococcal	Negative parent attitude regarding safety/necessity of vaccine	Educational intervention with 8-min video improved vaccine acceptance	Social desirability bias
Maltezou et al., 2009 [30]	1 year	Influenza	Lack of time and inconvenience	Lectures in hospital/mobile vaccination team visit-significant impact	No baseline data; no feedback
Fiks et al., 2013 [31]	1 year	HPV	Parental concerns, clinicians’ beliefs and practice concerns.	Combined interventions increased vaccination rates	Lack of large-scale study
Muehleisen et al., 2007 [32]	9 months	DTAP, HBV, HiB, IPV, MMR, Td	Lack of parental awareness	Increased reporting of immunization	Improper documentation/lack of prior immunization records, single-centric study
Ahlers-Schmidt et al., 2010 [33]	Not mentioned	General vaccine	Parental concerns about safety and lack of knowledge	Increased vaccine acceptability	Demographically not generalizable
Cates et al., 2011 [34]	6 months	HBV	Lack of awareness	Increase in vaccination acceptance and uptake	Socio-economic disparity in demographics
Pandey et al., 2011 [35]	Not mentioned	HPV	Inadequate information	Female students had better awareness; medical teaching had better impact	Single-centric study
**Media-based approach**					
Brown et al. 2015 [44]	Not mentioned	Routine vaccine	Not mentioned	60% mothers preferred immunization reminders by cellphones and SMS	Study not including rural population
Saville et al., 2014 [45]	4 months	General vaccine	Not mentioned	Preferred modality email or telephone	Socio-economic demography not generalizable
Hofstetter et al., 2013 [43]	3 months	General vaccine	Not mentioned	Text messages recall widely accepted	Socio-demographic data not generalizable
Kharbanda et al., 2009 [42]	Not mentioned	General vaccine	Not mentioned	Preferred method was text messages	Demographically not generalizable
Clark et al., 2015 [41]	Not mentioned	General vaccination	Not mentioned	Parents more willing to communicate by phone call	Lack of specificities
Lemstra et al., 2011 [40]	1 year	MMR	Low income	Limited additional benefits	Substantial study population not able to be contacted; incorrect telephone data
Milkman et al., 2011 [38]	1 month	Influenza	Lack of knowledge	Increased vaccination rate	Small sample size; single-centric study
Stockwell et al., 2012 [37]	6 months	Meningococcal (MCV4); tetanus diphtheria-acellular pertussis (Tdap)	Low income	Immunization reminders beneficial; increased vaccine uptake	Lack of sample size of parents recorded in cell phone registry
Zhang et al., 2019 [36]	Not mentioned	Acceptance of new target vaccination policy	Negative attitude towards immunization	Public figures/media messages can influence attitudes	Small study size. Did not identify demographic predictors
Garcia-Dia, 2017 [46]	3 months	Routine vaccine	Lack of awareness	Preference of text message along with Picture	Study conducted only in rural setting
Moniz et al., 2013 [39]	2 years	Influenza	Lack of awareness	Text messages not effective	Single socio-demographic group

### Community health trainings

Out of the total 33 studies considered, there were nine studies that were based on community health training strategy. Majority of the studies revealed parents/caregivers of children as the study population except for one study that primarily addressed the issue of vaccine hesitancy in religious leaders of a community. The most targeted vaccines were diphtheria pertussis tetanus (DPT1, DPT2, DPT3) vaccine, Bacille Calmette-Guerin (BCG) vaccine, poliovirus 3, measles, influenza, and HPV vaccine. Lack of knowledge, negative parental attitude, and misconceptions were the foremost encountered causes for vaccine hesitancy that were addressed predominantly by health workers/medical interns [15–19]. Home visits and information campaigns were the most common types of community training modalities except for the two studies that had personally controlled health management systems (PCHMS) and community-level nutrition information system for action (COLNISA) as community health training strategies that led to an overall rise in vaccine coverage from 21 to 33% [20, 21, 43, 44]. Community activity for systematic engagement of parents and home visits by community health workers and medical interns significantly improved program acceptance and utilization of immunization services (Table 2).

### Incentive-based approach

Five studies published between 2008 and 2013 were identified that focused on performance-based incentives for vaccination [24–28]. Incentive-based approach mostly involved general hospitals in the rural and lower socio-economic strata of the society. Most of these studies suggested monetary incentives only. Influenza, BCG, polio, DPT2, DPT3, measles, HBV, meningococcal 4 (MCV4), and tetanus diphtheria-acellular pertussis (Tdap) were the most sought-after targeted vaccines. A dearth of financial burden and negligence were the suggested reasons for vaccine hesitancy. Findings of these studies suggested that incentives had a high impact on the uptake of immunization services. The effect of non-financial incentives on vaccine uptake for parents and communities located in low-income settings (India) was moderate (RR: 2.16, [CI: 1.54, 2.78]) [25], except for one study that depicted no increase in vaccine acceptance using incentive-based search strategy [27] (Table 2).

### Technology-based health literacy

Lately, leveraging on the health literacy using technology such as informative posters, leaflets and videotapes, social media, organizing lectures, etc., were used to bring behavioral change regarding vaccination. The studies depicted that this intervention strategy was mostly acted upon in urban primary care practices and large multispecialty medical organizations. Inadequate information /rumors, parental concerns about safety and lack of awareness, clinicians’ beliefs and practice concerns attributed to vaccine hesitancy [18, 29–32]. The eight studies available highlighted and dealt with vaccine hesitancy towards polio vaccine, varicella, pneumococcal influenza, DTPDTP, hepatitis B (HBV), hemophilus influenza B (HiB), inactivated polio vaccine (IPV), and measles mumps rubella (MMR). These studies suggested that educational intervention using videos, posters, and lectures demonstrated an improved vaccine acceptance (Table 2) [33–35].

### Media engagement

Interventions such as reminder calls, SMS, and emails were adopted as media-based strategy in nine studies to address vaccine hesitancy. Most of the studies targeted general vaccines whereas only four out of eleven studies had interventions directed towards meningococcal (MCV4), tetanus diphtheria-acellular pertussis (Tdap), MMR, and influenza vaccines [37–40]. Low income, negative attitude towards immunization, and lack of knowledge were the most recorded reasons for vaccine hesitancy. The overall study outcome with this intervention strategy revealed that simple recall messages through SMS and email were preferred; however, these did not bring the desired change in overcoming vaccine hesitancy (Table 2) [41, 42, 44, 45].

### Risk of bias

Out of the 33 studies reported, 29 studies noted a high risk of bias and one study reported no risk of bias. The risk of bias is calculated on the basis of study design, analysis, withdrawals and dropouts, data collection practices, selection bias, invention integrity, blinding as part of a controlled trial, and confounders (Table 1).

## Discussion

The studies included interventions with diverse approaches that were implemented in different settings and targeted various populations, which helped us to get a holistic view of interventions globally to build confidence on vaccines, increase acceptance, and promote adequate immunization behaviors. In the review, we observed that the strategies suggested or evaluated were similar to traditional strategies such as IPC and social mobilization through education and empowerment, financial and non-financial incentives for motivation of beneficiaries and mobilizers, and technology assistance for communication to bring about a behavioral change. The studies used in this systematic review are equally from low, middle and higher-income countries focusing on involvement of political leaders, medical leaders, and mobile vaccination team for addressing the issues of vaccine hesitancy [30, 36].

Studies done by Fiks et al., Williams et al., Zhang et al., and Rahman et al. reported a lower risk of bias when compared to other studies, which could be due to variation in the study design and settings [17, 18, 31, 36].

Most of the interventions analyzed in the review were primarily either to inform or to educate the target population about the risks and benefits of vaccination using community health training strategy, as lack of knowledge or awareness about vaccines was observed to be the major cause of vaccine hesitancy. These studies reported effective improvement in vaccines uptake after the exercise. Two of these studies focused on the involvement of mothers for knowledge and experience sharing [15, 16]. A study conducted by Brugha et al. revealed a significant rise from 60 to 80% in vaccine coverage after 6 months of home-visit community health training program [16]. Involvement of mothers showed a significant improvement in vaccination coverage (33–85%) in another similar study done by Usman et al. [23]. Nine studies were based on parent-centered information or education about vaccination and social mobilization of parents by health workers/medical interns [15–23]. All these studies showed a significant impact in changing parents’ attitude towards their child’s vaccination. Messaging on vaccination from political and religious leaders also imparted a positive impact on vaccination uptake [17, 36]. A study conducted in Denver (USA) found significant difference in attitude and practices related to immunization among vaccine-hesitant and non-hesitant religious leaders [47]. Similarly, effective communication regarding polio vaccination with the community had shown positive impact in Nigeria [29]. However, variation in study sample with no consideration towards population dynamics was a potential limitation of all the nine studies from community health training theme, as some studies are conducted involving parents and caregivers [19, 23, 36]. In some studies, information is captured from children [21]. The sample sizes are also different for these studies as one of the studies involved more than thirteen thousand participants and while another study involved 117 participants [19, 22].

Findings of studies conducted by Mouzoon et al., Banerjee et al., and Stitzer et al. suggested that incentives had a high impact on the uptake of immunization services [24, 25, 27]. Conditional cash transfer program led to a huge increase in vaccination coverage resulting in 95% coverage along with incentive-based interventions were also found to be effective in rural Nicaragua. The study shows an increase of 10% in vaccination coverage rate among 12–23 months old children to 95% for DPT3 in treatment group as compared to 85% in the control group [26]. It was evident from the synthesis that the incentive-based strategies had a positive impact on bringing about vaccination acceptance. The benefit of incentive-based health promotions had always been significant but sustainability and adherence after intervention was debatable [28]. An increasingly popular strategy in health policy is the use of “incentive” to individuals to avoid health risks. In particular, we must ask whether incentive schemes are more effective than policies that aim to address directly the barriers to “healthy” behaviors, especially those existing among disadvantaged groups. Furthermore, the implementation of incentives in large populations remained a challenge. At the same time, integration of incentives with other mother and child health services such as the Janani Suraksha Yojana implemented by Govt. of India can bring a positive change in improving immunization uptake along with education on delivery and nutrition in low-income and low-education settings [25].

Gaps in awareness such as complete absence of knowledge, less knowledge, and misconceptions were known to be the principal factors for lack of adequate health-seeking behavior. Strategies focusing on behavior change through knowledge and awareness will be most suitable for complex behavioral dynamics as it targets multiple layers of decision-making—individual, family, and society [29]. Additionally, the benefits of health literacy using technology to bring about public awareness are not only multi-faceted but also have potential to change the whole health-seeking behavior paradigm and not just the behavior towards vaccines [18]. Using mobile technology and social media has also improves peoples’ awareness for managing health and service delivery [48].

Recently, educational videos, lectures in hospital settings, mobile vaccination team visits, social marketing, and web-based questionnaires have been used to bring about a behavioral change regarding vaccination. A study conducted in the rural areas North Carolina of (USA) using social marketing campaign raised the awareness among parents and reduced barriers in accessing the HPV vaccine successfully [34]. Similarly, HPV vaccination rates were 2% higher among 9–13-year-old girls within 6 months of campaign launch [34]. Evaluation of social media interventions by Muehleisen et al. (2007) and Lemstra et al. (2011) showed a positive effect on uptake of MMR vaccine [32, 40]. In Northern Nigeria, a relative increase of ~ 310% in the polio vaccination uptake was observed through an educational intervention with a video containing awareness message about polio vaccination [21].

Furthermore, the intervention focusing on the engagement of various kinds of media to reach the population has also proved to be efficient in creating awareness and promoting beneficial health-seeking behaviors [18]. Therefore, in conjunction with awareness-creating strategies, utilization of mass media in various forms such as print, audio, television, and social media can stimulate a positive perception among the population in different settings [21, 33]. However, improper documentation and socio-economic disparity in demographics was the major downside in the health literacy using technology-based intervention strategy.

Among all the strategies, recall strategies showed least improvement in mobilizing people from negative perception to acceptance. Furthermore, findings from a study in USA showed that parents aged 30 years and above preferred e-mail reminders as compared to other modes such as phone calls and text messages [43]. Few studies from USA and Nigeria have revealed a wide support and acceptability of text messages or SMS as a mode of immunization reminder or recall. A large proportion of parents had also shown willingness to be reminded about vaccinations by their health departments and via novel modalities such as email or text messaging [41, 45, 47]. Urban parents preferred reminders from their child’s doctor (46.7%) as compared to rural parents (33.7%) [37].

Although the recall strategies showed improvement in vaccine uptake by addressing the issues of vaccine hesitancy, they were inconsistent in all studies [40, 42, 43]. Therefore, it can be perceived that these kinds of passive reminders sent through modern communication channels may be only effective in case of technology-friendly populations. It is unlikely that mere recall messages through SMS or email, which were found to be preferred, will bring a desired change in the confidence on vaccines [38].

In light of the above knowledge, it is difficult to predict the superiority of any intervention over the other. Therefore, more studies with a better study design and targeting specific populations are required. Another reason for the lack of literature can be our limited access to indexing databases, which severely limits our capability to extract large amount of published literature.

## Conclusions

Vaccine hesitancy not only increases an individual’s risk of contracting a disease but also increases the risk for the community. Vaccine hesitancy is a complex issue, and no standalone strategy can address it. Despite the complexity of vaccine hesitancy and the broad range of its determinants, increasing awareness about benefits of vaccination, social media engagement activities, and carefully tailored strategies addressing the determinants of the hesitancy can bring about the desired change.

## Supplementary Information

**Additional file 1.**

## Data Availability

Presented in the manuscript; any additional data can be sent if requested, specifically.

## Abbreviations

AEFI: Adverse events following immunization
BCG: Bacille Calmette-Guerin
CI: Confidence interval
COLNISA: Community-level nutrition information system for action
DPT: Diphtheria pertussis tetanus
DTaP: Diphtheria, tetanus and pertussis vaccines
EPHPP: Effective Public Health Practice Project
HBV: Hepatitis B virus
Hib: Hemophilus influenza B
HPV: Human papillomavirus
IPV: Inactivated polio vaccine
MCV: Meningococcal vaccine
MMR: Measles mumps rubella
PCHMS: Personally controlled health management systems
PICO: Participants, Interventions, Comparisons, Outcomes
PRISMA: Preferred Reporting Items for Systematic Reviews and Meta-Analyses
RR: Relative risk
SMS: Short Message Service
Tdap: Tetanus diphtheria-acellular pertussis
TV: Television
UNICEF: United Nations International Children’s Emergency Fund
USA: The United States of America
WHO: World Health Organization
