# Supplementary material for: Strategies to overcome vaccine hesitancy: a systematic review
Source: Syst Rev. 2022 Apr 26;11:78. doi: 10.1186/s13643-022-01941-4 (PMC9044888; doi:10.1186/s13643-022-01941-4)
Supplement: Supplementary file 1 — Additional file 1. [file 13643_2022_1941_MOESM1_ESM.docx]

**The PRISMA for Abstracts Checklist**

| **TITLE** | **CHECKLIST ITEM** | REPORTED ON PAGE # |
| --- | --- | --- |
| 1. Title: | Identify the report as a systematic review, meta-analysis, or both. | 1 |
| **BACKGROUND** |  |  |
| 2. Objectives: | The research question including components such as participants, interventions, comparators, and outcomes. | 2-3 |
| **METHODS** |  |  |
| 3. Eligibility criteria: | Study and report characteristics used as criteria for inclusion. |  |
| 4. Information sources: | Key databases searched and search dates. | 3-6 |
| 5. Risk of bias: | Methods of assessing risk of bias. |  |
| **RESULTS** |  |  |
| 6. Included studies: | Number and type of included studies and participants and relevant characteristics of studies. |  |
| 7. Synthesis of results: | Results for main outcomes (benefits and harms), preferably indicating the number of studies and participants for each. If meta-analysis was done, include summary measures and confidence intervals. | 6-8 |
| 8. Description of the effect: | Direction of the effect (i.e. which group is favoured) and size of the effect in terms meaningful to clinicians and patients. |  |
| **DISCUSSION** |  |  |
| 9. Strengths and Limitations of evidence: | Brief summary of strengths and limitations of evidence (e.g. inconsistency, imprecision, indirectness, or risk of bias, other supporting or conflicting evidence) | 8-12 |
| 10. Interpretation: | General interpretation of the results and important implications |  |
| **OTHER** |  |  |
| 11. Funding: | Primary source of funding for the review. |  |
| 12. Registration: | Registration number and registry name. | Title page |
